# Intersectional inequalities in science

**DOI:** 10.1073/pnas.2113067119

**Published:** 2022-01-04

**Authors:** Diego Kozlowski, Vincent Larivière, Cassidy R. Sugimoto, Thema Monroe-White

**Affiliations:** ^a^Faculty of Science, Technology and Medicine, University of Luxembourg, Esch-Sur-Alzette 4364, Luxembourg;; ^b^École de Bibliothéconomie et des Sciences de L’information, Université de Montréal, Montreal, QC H3T 1N8, Canada;; ^c^Observatoire des Sciences et des Technologies, Université du Québec à Montréal, Montreal, QC H3C 3P8, Canada;; ^d^Department of Science and Innovation-National Research Foundation Centre of Excellence in Scientometrics and Science, Technology and Innovation Policy, Stellenbosch University, Stellenbosch 7602, South Africa;; ^e^School of Public Policy, Georgia Institute of Technology, Atlanta, GA 30313;; ^f^Campbell School of Business, Berry College, Mount Berry, GA 30149

**Keywords:** intersectionality, science of science, bibliometrics, race, gender

## Abstract

The US scientific workforce is primarily composed of White men. Studies have demonstrated the systemic barriers preventing women and other minoritized populations from gaining entry to science; few, however, have taken an intersectional perspective and examined the consequences of these inequalities on scientific knowledge. We provide a large-scale bibliometric analysis of the relationship between intersectional identities, topics, and scientific impact. We find homophily between identities and topic, suggesting a relationship between diversity in the scientific workforce and expansion of the knowledge base. However, topic selection comes at a cost to minoritized individuals for whom we observe both between- and within-topic citation disadvantages. To enhance the robustness of science, research organizations should provide adequate resources to historically underfunded research areas while simultaneously providing access for minoritized individuals into high-prestige networks and topics.

## Significance

The US scientific workforce is not representative of the population. Barriers to entry and participation have been well-studied; however, few have examined the effect of these disparities on the advancement of science. Furthermore, most studies have looked at either race or gender, failing to account for the intersection of these variables. Our analysis utilizes millions of scientific papers to study the relationship between scientists and the science they produce. We find a strong relationship between the characteristics of scientists and their research topics, suggesting that diversity changes the scientific portfolio with consequences for career advancement for minoritized individuals. Science policies should consider this relationship to increase equitable participation in the scientific workforce and thereby improve the robustness of science.

Strong disparities are observed in the composition of the scientific workforce. At the global level, women account for less than a third of scientists and engineers (1); a percentage that is similar to their proportion of scientific authorships (2). In the United States, women represent 28.4% of the scientific workforce, and this percentage varies by domain, with a high of 72.8% in psychology and a low of 14.5% in engineering (3). Disparities are also observed at the intersection of race and gender, with White men comprising a disproportionate amount of the US workforce (4). Although the trend is changing—faculty of color increased from 20% of the scientific workforce in 2005 (5) to 25% in 2018 (6)—increases have not been observed equally across all racially minoritized groups. For instance, the proportion of Black (5 to 6%) and American Indian/Alaska Native (1%) scholars remained relatively stable, while Latinx representation nearly doubled (3.5 to 6%), and Asian representation increased from 9.1 to 11%. Gender differences are also observed within racial categories: men account for a higher share than women, especially for White and Asian/Pacific Islanders (6). Likewise, the presence of minoritized groups varies substantially by discipline. Science, Technology, Engineering/Computer Science, and Mathematics (STEM) disciplines exhibit less demographic diversity than non-STEM fields (7). For example, in Biology, only 0.7% of faculty identify as Black, despite representing 12.2% of the US population (7).

These differences characterize the unequal representation of populations within the scientific community. Such disparities are often a manifestation of inequality—unequal outcomes—and inequity—the degree to which these outcomes are a result of impartiality or bias in judgement. Women and other minoritized populations are underrepresented in scientific publishing (8, 9), for example, and this can be associated with unequal outcomes in peer review (10, 11). Inequalities have been observed at several other pivotal evaluation points in science, including applications for laboratory manager positions (12), grant submissions (13, 14), and scholarly impact (2). Several forms of implicit bias may contribute to these inequities: from perceptions of brilliance (15) to gendered scripts on women’s commitment to science (16). Overt forms of discrimination found in other spheres of society are also observed within the scientific community, such as stereotypes about gender and race (17), anti-Black institutional policies (18), and structural racism (19, 20).

Studies that examine inequities and inequalities at the individual level are often anchored in a justice perspective (21), whereby scientific principles such as universalism (22) are tested against the current system, thus challenging the conception that science is a meritocracy. In contrast with studies of individual-level success, utilitarian studies focus on collective gains and test whether higher equity improves the robustness of science. Extant studies have demonstrated that racial diversity leads to increased productivity (i.e., sales and profits) in industry (23) and that diverse groups outperform homogeneous ones in cognitive tasks (24). In science, diversity in the composition of scientific teams has been linked to higher citations (25) and tied to gains in innovation (26). This emergent body of literature suggests that there are scientific and societal benefits to increasing diversity in science. Studies should, therefore, consider the rich interplay between social identities and scientific work.

A growing body of work examines the affinity between social identities and topic selection. For example, in medical research, decades of male dominance led to little attention to sex differences in medicine (27, 28). The changing demographics of the research community improved the situation, as women are more likely to include female subjects (29) and to report sex as an analytical variable (30). Women are also more likely to produce scientific discoveries that lead to women’s health patents and to contribute to patenting in this area (31). Funding—one of the main drivers of research activity—is similarly affected by researchers’ social identifies and align with topic selection. For example, funding outcomes at the NIH were found to be lower for Black and African American applicants. This was largely explained in topic selection: These investigators were more likely to propose research on topics with lower success rates (e.g., human subjects research and research on health disparities) relative to White and Asian investigators (32). These outcomes have implications for innovation and scientific competitiveness: Racialized and gendered groups are more likely to contribute novel scientific contributions, yet their work is often neglected by other scientists (26). Taken together, these studies suggest that unequal representation in science leads to underinvestigation of particular topics and may serve to stymie innovation. This motivates a more nuanced understanding of barriers to success for minoritized populations and how these observed disparities intersect with complex social identities, fine-grained topic selection, and the reward structure of science.

Intersectionality was initially introduced as an analytic framework for understanding how interrelated and mutually shaping categories of race and gender served to compound inequalities for minoritized women (33–36). These studies emphasize women’s racialized and gendered experiences by explicitly situating minoritized women as central actors in power struggles and social inequalities (23, 33, 37–41). The intersectional framework has since been expanded and used to frame the marginalization experienced by minoritized groups at the intersection of race, gender, sexual orientation, class, and other identities (42). While gender inequities and inequalities have been the focus of several recent large-scale analyses (2, 10, 43–46), very few studies have focused on the racial and ethnic composition of authors (46–49). Studies from an intersectional perspective (e.g., women of color) have been predominantly qualitative, based on self-reports, or focused on a particular field or set of subfields (50–52). These studies provide rich evidence of the impact of structural biases on career trajectories through valuable storytelling and suggest a need for an intersectional lens to large-scale studies. Furthermore, these studies reveal that a failure to disaggregate at the intersection of race and gender may obfuscate novel findings and lead to the generation of overly simplistic insights and policy recommendations (47).

Therefore, we seek to interrogate the space between the composition of the scientific workforce and the topical profile of science from an intersectional perspective. This study extends investigations of gender disparities in science by providing a macrolevel of view of the phenomena that accounts for the intersection of race, gender, and topic. Our focus on the United States enables us to contend with the unique contextual factors that have led to disparate representation between genders and racial groups in science. Despite the acknowledged importance of race and gender as factors of inequality and decades-long policy interventions, there remains a paucity of evidence on how the selection of fields and topics is scattered across groups at a detailed level and the relation between topics and scientific impact. This paper attempts to demonstrate that the election of the object of study is related to race and gender, with implications for scientific progress and the evaluation of scientists.

## Materials and Methods

We examine the publication patterns of US-affiliated first authors between 2008 and 2019. Our data consist of 5,431,451 articles indexed in the Web of Science (WOS) database and 1,609,107 distinct US first authors. We focus on first authors, as they are generally those who have contributed the most to an article (53, 54) and represent the most visible name in bibliographic references. The metadata includes authors’ given and family names, which are used to infer race and gender. Authors were disambiguated using the algorithm developed by Caron and van Eck (55). The gender disambiguation algorithm builds on the method presented in Larivière et al. (2), which uses census data and country-specific lists of men and women names to assign probable gender to given names and, in the case of certain countries (e.g., Russia and Ukraine), family names. Gender is considered in a binary way, as other genders can only be assigned through self-identification. This is an acknowledged limitation of the study.

Racial categories are a country-dependent social construct, and not all countries have such categorizations. Therefore, our analysis focuses on the specific cultural construct of race found in the United States. For the inference of race/ethnic origin, we use the 2010 US Census information on family names and racial groups (56). Racial groups considered in the US Census are the following: 1) Non-Hispanic White Alone (White), 2) Non-Hispanic Black or African American Alone (Black), 3) Non-Hispanic Asian, Native Hawaiian, and Other Pacific Islander Alone (Asian),* 4) Non-Hispanic American Indian and Alaska Native Alone (AIAN), 5) Non-Hispanic two or more races (two or more), and 6) Hispanic or Latino origin (Latinx).^†^ Given that AIAN and two or more account for only 0.69% and 1.76% of WOS authors, respectively, they were removed from the analysis. Census data provides the number of people that identify with each racial group for the 162,253 most common family names. Using family names, we compute each author’s associated probability to each racial group, instead of assigning the most probable group, and using these probabilities to compute weighted aggregates, in which each author contributes to each group’s aggregate as a function of the racial group distribution associated with its family name. This means, for example, that when computing the average citations by race, we assign the citations of an article fractionally to each group according to the corresponding distribution. In other words, we do not assign authors to a unique racial category. In previous work (58), we have shown that, given the overlap of Black and White family names (59, 60), the use of a threshold—filtering those names with a probability for a single group above a threshold and assigning all authors with that name to that single category—underestimates the proportion of Black authors. This distinction is critical: We do not aim to identify each author’s self-perceived racial category but to build aggregates of racial group disparities. For those names that do not appear in the census, we impute the mean distribution in the subset of authors used at each point in the analysis. For a detailed description of the racial inference methods, see ref. 58, *SI Appendix*), as well as the accompanying website.^‡^

Fields and subfields are defined according to the journal classification developed for the US NSF (61). Following ref. 62, we used articles’ abstract, title, and keywords to train a latent Dirichlet allocation (LDA) model to infer the topics within a corpus of papers and the distribution of topics within each article. LDA is an unsupervised model that assumes that there is a fixed number of topics within the corpus that correctly describes its content. Each topic is defined as a distribution over words, and we use the top five words from each topic to infer its semantic content. Given an article’s topic, there are some words that are more likely to repeat than others; LDA provides the list of most repeated words for each topic, and we use those to infer topicality. The objective of this model is to create research topics as detailed as the sampling allows, implying a trade-off between granularity and repetition of topics. LDA models are performed on groups of disciplines to identify topics with an independent meaning (63). Given the sample size in Social Science and Health and the interpretability of results across different experiments, we found the optimal number of topics for our analysis to be 200 for Health and 300 for Social Sciences. Using manual inspection, a higher number of topics in each case led to the repetition of words between topics, while fewer topics led to less detailed results. For a selected group of topics, we defined a single label based on these top words. See *SI Appendix* for an explanation of the robustness analysis of the LDA model.

Scholarly impact is assessed through field- and year-normalized citations (64), using an open citation window covering publication years through the end of 2019. For each article, we infer the first authors’ gender and distribution over racial categories as well as over topics. Each article then has a probability distribution over racial categories, a binary classification over gender, and a probability distribution over topics. Aggregate results are obtained using fractional counting over these three dimensions. For example, an article can have a first author whose name has a 0.7 racial classification probability of being a Black author, a 0.3 of being a White author, and whose gender is inferred to be woman. It also has a 0.8 probability on topic A, 0.1 on topic B, and so on. Therefore, this article contributes an additional 0.56 (0.7 × 0.8) authors to the group of Black women in topic A, 0.07 (0.7 × 0.1) authors to the group of Black women in topic B, and so on across all topics and racial groups for women. The weighted sum over these dimensions, plus the citations, gives us the aggregate results of the distribution over topics by race and gender and the average number of normalized citations per topic, race, and gender. Over- and underrepresentation in topics of racial groups and genders are based on the overall proportion that each group represents across all articles combined.^§^

## Results

**Fig. 1.**
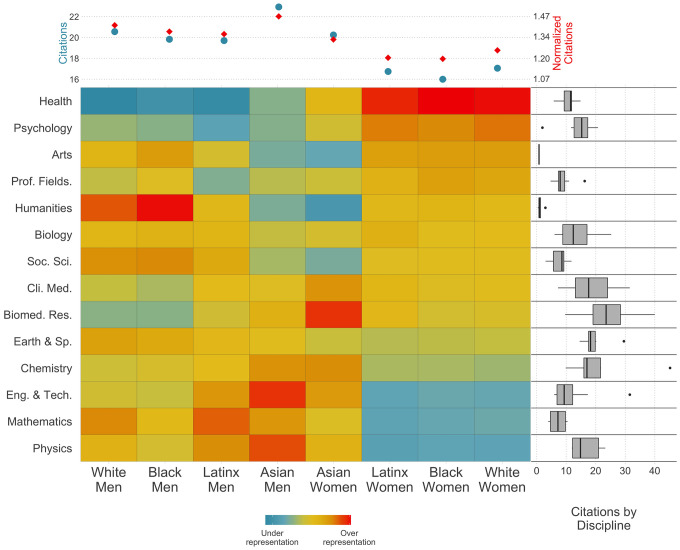
Scholarly impact and distribution of race and gender of authors by field. Average number of raw and field-normalized citations by group. Over- and underrepresentation of groups by discipline, with respect of their average proportion in all fields. Distribution of the average number of raw citations by specialty within each discipline; the hinges of the box correspond to the first and third quartiles, the whiskers extend to the lowest values no further than 1.5 time the interquartile range (IQR) from the hinge, and dots represent values further than 1.5 times IQR. Data consist of US first authors within the WOS from 2008 to 2019. Racial categories from the census corresponding to AIAN and two or more were excluded from the racial inference because of lack of data. On the vertical axis, fields are sorted by the relative over/underrepresentation of Black women authors. On top, we show the average number of citations by group, while on the right, each boxplot summarizes the distribution of citations of all papers published in those fields.

Comparison of race and gender demographics of US first authors with that of the US population shows that White and Asian populations are overrepresented among US authors, while Black and Latinx populations are underrepresented (*SI Appendix*, Fig. S1). Relative representation varies by field (Fig. 1). Black, Latinx, and White women exhibit similar representation: They are highly underrepresented in Physics, Mathematics, and Engineering and overrepresented in Health (*SI Appendix*, Figs. S2 and S3), Psychology, and Arts. Asian women follow a different pattern, with underrepresentation in Arts, Humanities, and Social Sciences and overrepresentation in Biomedical Research, Chemistry, and Clinical Medicine. Black, Latinx, and White men are underrepresented in Psychology and Health, together with Asian men, but this latter group is also underrepresented in Humanities and Social Sciences and overrepresented in Physics, Engineering, Math, and Chemistry. Men first authors are generally more cited than women, and Asian authors are more cited than Black, Latinx, and White authors, both in raw citations and field-normalized citations. For White, Black, and Latinx women the citation gap reduces when considering normalized citations, showing that they are more present in lower-cited fields. Nevertheless, even when considering field-normalized citations, the gap remains.

**Fig. 2.**
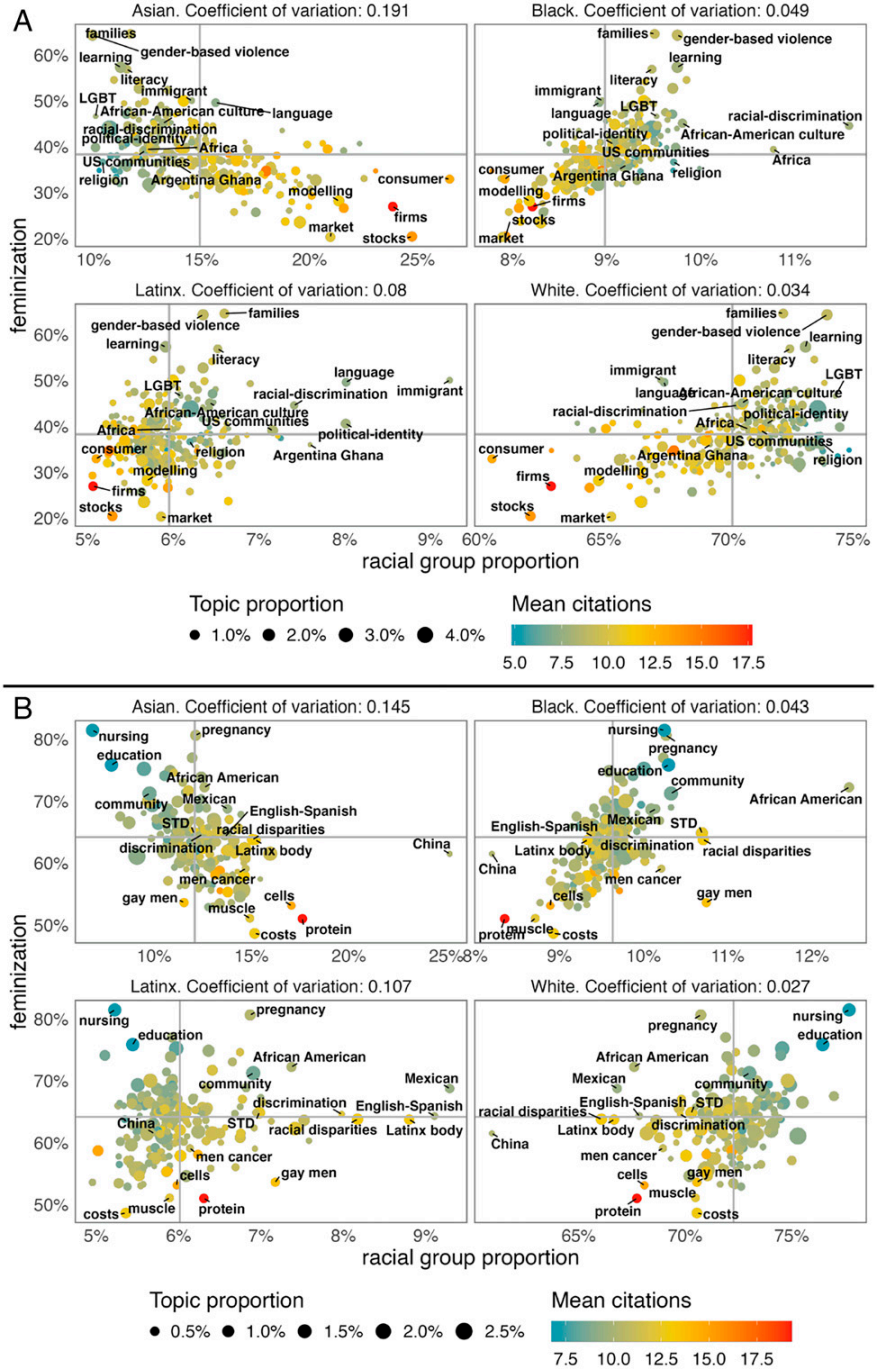
Distribution of topics by racial group and gender participation. (*A*) Social Sciences, Humanities, and Professional Fields, (*B*) Health. For Social Sciences, Humanities, and Professional Fields (*n* = 283,589 articles), we train an LDA model for 300 topics. For Health (*n* = 142,032), we train the LDA model for 200 topics. The vertical axis shows the proportion of women, while the horizontal axis shows the proportion by each racial group. In color, there is the mean number of citations by topic. The CV, as a standardized measure of variability, is provided for each racial group. Topics with the highest proportion in each race and in each gender are highlighted (labeled). Racial categories from the census corresponding to AIAN and two or more were excluded from the racial inference because of lack of data. Minimum number of average citations: 4.37, max. 15.74, and mean 9.25. STD, sexually transmitted disease.

To better understand and explain intersectional differences in citations, we explore the role of research topics for disciplines in the Humanities, Social Sciences, Professional Fields, and Health.^¶^ Fig. 2 presents feminization—i.e., the proportion of women authors of each topic (*y*-axis)—by racialization—i.e., the proportion of authors from a racial group in each topic (*x*-axis)—for Social Sciences (Fig. 2*A*)^#^ and Health (Fig. 2*B*).^ǁ^ The color of each node (topic) provides the mean number of citations, while size represents relative importance in the dataset.

In the Social Sciences, topics with the highest proportion of Asian authors are related to topics in economics and logistics, like stocks, consumers, firms, and market. These topics are also those where White and Black authors are least represented. Black authors are highly represented on topics of racial discrimination, African American culture, and African studies and communities. Religion is one of the few topics in which both Black and White men are overrepresented. Latinx authors are highly represented in topics related to immigrants, political identity, and racial discrimination, the latter of which is also shared with Black authors. Black and Latinx authors perform research on topics specific to language literacy, as well as on African and Latin-American countries, respectively. Latinx authors publish on topics associated with Latin-American issues and those that redefine the Latinx identity within the United States. Of particular interest is the topic of language literacy, which is both highly feminized and highly Latinx and constitutes a mixture of a traditional gender role (teaching, related with reproductive labor) and the learning of a second language, a topic that is highly relevant to migrant communities.

Fig. 2 also shows the coefficient of variation (CV) for each racial group’s proportion on topics. A high CV means that the group has a high participation on some topics and a small participation on others, relative to its average proportion. Asian authors present the highest CV, while White authors exhibit lowest. This suggests that Asian authors are highly specialized, focusing on certain topics, while White authors are present in a wider range of topics. Black and Latinx authors show greater specialization, focusing on a smaller number of topics. White authors are more evenly distributed among topics; however, this is expected, as they account for the majority of the author population. The most highly feminized topics include gender-based violence, families, learning, and lesbian, gay, bisexual, and transgender (LGBT) studies. These results implicate a relationship between traditional gender roles, and topics that relate specifically with gender-based identity and inequality, and to nonhegemonic gender representations (i.e., gender expressions that do not correspond to binary male/female categorizations).

Several important topics appear at the intersection of race and gender. Because of space constraints, it is not possible to assign a label for all topics in Fig. 2; however, the accompanying website presents an interactive visualization displaying topics along the diagonal of Quadrant I that are both highly feminized and racialized. Among Black women authors, for example, we find topics such as black women violation (topic 122), equality promotion (topic 149), and social identity (topic 210). Among Latinx women authors, in addition to topics on language, we find residential segregation (topic 103), gender gap and international migration (topic 300), social class (topic 64), and global south (topic 225), among others.

In Health, the topics with the highest representation of Asian authors are China, proteins, cells, and the economics of health (i.e., costs). Black authors publish on topics about racial disparities and sexually transmitted diseases—with a special emphasis on African Americans among Black women and gay men for Black men. This latter topic is also relevant for Latinx men. Latinx authors publish more on topics that mention the Latinx population, racial disparities, (a topic that is shared with Black authors), and English-Spanish, a topic similar to that which was previously found in the Social Sciences. The CV between topics by racial group also shows that Asian authors are the most specialized, followed by Black and Latinx authors, and White authors are the most ubiquitous across topics. The most feminized topics are about nursing, pregnancy, and education, reinforcing the association of women with care- and service-related research (65, 66).

### Scholarly Impact by Topic.

**Fig. 3.**
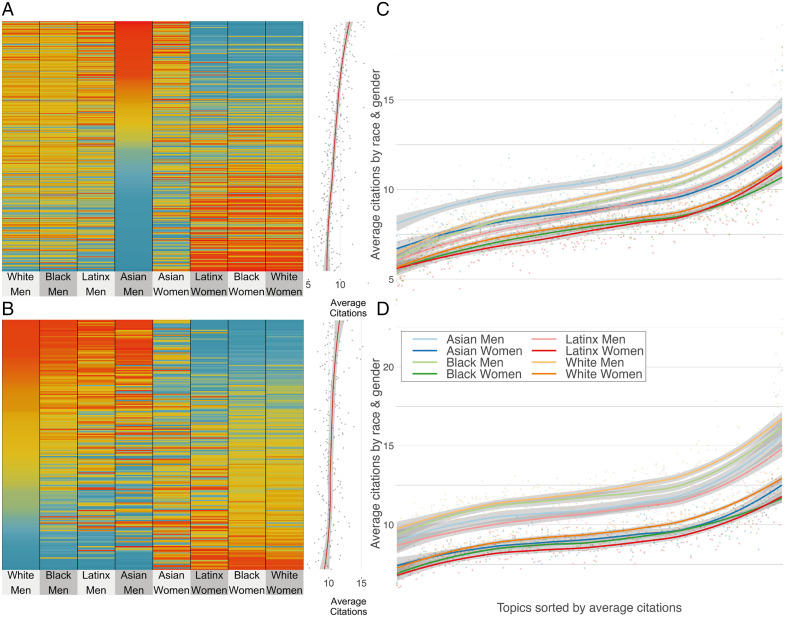
Scholarly impact by topic. Over- and underrepresentation of groups by topic, in Social Sciences (*A*) and Health (*B*). Topics sorted by the participation of the most cited group in each case. On the right margin, the figure shows the number of citations by topic, and loess smoothing. Distribution of average topic citations by race and gender in Social Sciences (*C*) and in Health (*D*). Topics are sorted by average number of citations, and a smoothing function is drawn for each group using loess to model the evolution of the expected number of citations, as the topics become more cited on average. The gray shadow in each model represents the 95% confidence level, and therefore, when these shadows do not overlap, the differences between groups are significant. Racial categories from the census corresponding to AIAN and two or more were excluded from the racial inference because of lack of data.

Our results demonstrate that macrolevel differences in citation rates are observed at the intersection of race and gender—even when controlling for disciplines (Fig. 1)—and that topic selection is related to author race and gender (Fig. 2). It stands to reason, therefore, that there might be a relationship between the populations engaged in certain topics and the citation density of these topics. Fig. 3 presents the over- and underrepresentation of race and gender of authors by topic, sorted by the participation of Asian men in Social Sciences (Fig. 3*A*) and by White men in Health (Fig. 3*B*), the two most highly cited groups in each discipline. The average number of citations of a topic is positively correlated with the presence of Asian and White men. Fig. 3 *C* and *D* provides the average number of citations by race and gender within each topic** for each discipline, respectively. In the Social Sciences, Asian men have a higher number of citations; they tend to be more present within highly cited topics and are more cited than other groups within lower-cited topics. All other groups start with a relatively similar number of citations, which later split into three branches. White and Black men increase their relative number of citations to equalize those of Asian men in the highest cited topics. Latinx men and Asian women follow a similar course but yield fewer citations for the highest cited topics. Black, White, and Latinx women form a block with systematically fewer citations than all other groups. Health presents a stronger gender split: Men, regardless of racial categorizations, are significantly more cited along the distribution of topics, with White and Black men having slightly more citations than Asian and Latinx men. Women from all racial groups present a lower number of citations, with White women presenting a slightly higher number of citations for highly cited topics than Asian, Latinx, and Black women. This provides evidence of the intersectional between- and within-topic disadvantage for minoritized groups: 1) minoritized groups are overrepresented in lowly cited topics and underrepresented in highly cited topics; and 2) their work is less cited within and across topics, especially where they are underrepresented.

## Discussion

Inequalities in science have been studied for a century (67, 68), and several analyses have shown that these inequalities are the consequence of a nonmeritocratic scientific system (69–71). Our results show that minoritized authors tend to publish in scientific disciplines and on research topics that reflect their gendered and racialized social identities. Specifically, we have shown a contrast between the topic specialization of US Asian, Black, and Latinx first authors, reflected via a higher CV, and the ubiquity of White authors. The even participation of White authors across topics shows that the relation between race and research topic operates primarily on minoritized authors. In other terms, there is a privilege of choice in scientific knowledge production, wherein research on a particular topic is influenced by scientist’s race and gender. As Bourdieu explains, the amount of scientific capital possessed by a researcher defines the strategies they can follow (72). The ubiquity of White men in science and across topics implies that this demographic group has a wider range of possible strategies to follow and an advantage in the way their scientific capital can be invested, reinforcing inequalities in scholarly outcomes.

We found that differences in research impact can be at least partially explained by topics’ citation density but that within-topic differences remain. The compound effect of different citation rates of topics and unequal distribution on topics by race and gender leads to negative effects for marginalized groups and for science itself, as some topics become systematically less studied. The history of science is ripe with examples of understudied topics, such as female genitalia, which had direct implications on the life expectancy of women (73). Assuming constant productivity (74) and considering the career age of authors, we can estimate the cumulative loss in particular topics over the last 40 y, assuming that researchers with 20 y of publication activity produced 20 times that of incoming researchers. If the author distribution over the last 40 y would have matched the 2010 US Census, there would have been 29% more articles in public health, 26% more on gender-based violence, 25% more in gynecology and in gerontology, 20% more on immigrants and minorities, and 18% more on mental health (*SI Appendix*, Figs. S4–S6). While this counterfactual scenario is coarse, it highlights the fact that a different body of knowledge would be produced in the absence of inequalities and that this body would more closely reflect the spectrum of topics relevant across society. The diversification of the scientific workforce is necessary to create a scientific system whose results benefit all of society.

This paper has provided evidence of the relation between race, gender, research topic, and research impact and contributes to the wider dialogue on intersectional inequalities in science. However, race and gender are not the only spaces of inequality in science; several other variables should be included to create a fully intersectional understanding of inequalities in science. Socioeconomic status, when intersected with race, gender, and topic, is likely to have large effects: A recent study suggested that the estimated median childhood income among faculty is 23.7% higher than that of the general population (75). Inequities have also been observed on the basis of disability (76) and sexual orientation (77)—variables that are often excluded or underreported in studies of the scientific workforce. Attrition and career age (78, 79) may also play an important role here, as well as the prestige of institutional affiliations (80, 81). Causal modeling that considers topic choice, along with markers of prestige, would be germane in understanding the different mechanisms through which systemic inequalities are mediated. Finally, racial categories used in this research are only meaningful in the context of the US academic workforce; further research should be performed to understand general patterns across the globe and provide insights on the role of science policy in mitigating disparities.

Discrimination defies notions of objective, apolitical, and meritocratic ideals in scientific discourse (21); a perception that serves to reinforce and mask race and gender biases in science (81). Structural racism (82) remains a persistent source of mental and physical strain on minoritized groups in the United States (83–87), whose calls for justice across socioeconomic (e.g., healthcare, housing, education, finance, and criminal justice) and professional domains are intermittently elevated (and subsequently ignored) in accordance with the ebbs and flows of American racial discourse (88). Academia is no different in this regard (89, 90). The underrepresentation in science is similar to other sectors and may be attributed to the pervasive legacy of US federal- and state-sanctioned campaigns of systemic, racialized exclusion aimed to reduce the representation and participation of minoritized race groups in all aspects of human life (12, 17, 19, 91–94). Recent calls for increased transparency and accountability in graduate student recruitment, retention, and faculty hiring and promotion (95, 96) are particularly notable after the marked increase in media attention on anti-Black police violence, the Black Lives Matter movement, and the disproportionate impact of the COVID-19 pandemic on Black and Latinx populations (97–100) and on women academics (101–103). The effect of related policy interventions in response to these events remains to be tested.

Our analysis suggests structural effects that reproduce systemic inequities in terms of value assigned to particular topics, in both scientific evaluation and distribution of resources. Several policy recommendations emerge from this analysis. First, scientific institutions need to recognize the existence of knowledge gaps related with author race and gender segregation and promote topics in which gendered and racially minoritized authors are more present. Funding agencies can take immediate action to allocate increased funding in areas that have been historically underrepresented (31). Such funding will affect the entire academic reward system: Funding is strongly correlated with productivity and impact, both of which are associated with institutional advancement and rewards (104, 105). This has implications for individual scientists but also serves to increase the visibility of and participation in understudied areas. Second, institutions need to promote diverse participation within high-impact topics, taking into account the need for resources and initiatives that provide access for marginalized populations into high-prestige networks. Taken together, these activities will serve to both reduce the variance in impact across topics and reduce the within-topic disparities at the intersection of race and gender, thereby increasing equity in science and expanding the knowledge horizon.

## Supplementary Material

Supplementary File

## Data Availability

Detailed methods and results tables and code and materials data have been deposited in the University of Luxembourg website (https://sciencebias.uni.lu/app/) and GitHub (https://github.com/DiegoKoz/intersectional_inequalities). All the data for this article are available in the supporting information. Restrictions apply to the proprietary bibliometric data, which is used under license from Clarivate Analytics. To obtain the bibliometric data in the same manner as the authors (i.e., by purchasing them), readers can contact Clarivate Analytics (https://clarivate.com/webofsciencegroup/solutions/web-of-science/contact-us/).

## Footnotes

*Per 2010 US Census classifications, “Asian” refers to a person with origins East Asia, Southeast Asia, or the subcontinent of India; meanwhile, Native Hawaiian and Other Pacific Islander refers to a person having origins in Hawaii, Guam, Samoa, or other Pacific Islands.

^†^The authors use the term Latinx as a gender-neutral term, consistent with its perceived meaning, according to the 2019 National Survey of Latinos (57).

^‡^https://sciencebias.uni.lu/app/.

^§^See the *SI Appendix* for how we operationalize over- and underrepresentation.

^¶^The analysis was also carried out for all fields: https://sciencebias.uni.lu/app/.

^#^*SI Appendix*, Table S1 provides the top words of each of these topics. An interactive version of this plot, with labels for all topics is available at https://sciencebias.uni.lu/app/.

^ǁ^*SI Appendix*, Table S2 provides the top words of each of these topics. An interactive version of this plot, with labels for all topics is available at https://sciencebias.uni.lu/app/.

**An interactive version of this visualization with information on each topic and group is available at https://sciencebias.uni.lu/app/.
