# Supplementary material for: Intersectional inequalities in science
Source: Proc Natl Acad Sci U S A. 2022 Jan 4;119(2):e2113067119. doi: 10.1073/pnas.2113067119 (PMC8764684; doi:10.1073/pnas.2113067119)
Supplement: Supplementary File [file pnas.2113067119.sapp.pdf]

PNAS
www.pnas.org

Supplementary Information for
Intersectional inequalities in science

Authors: Diego Kozlowski, Vincent Larivière, Cassidy R. Sugimoto, Thema Monroe-White*

*Corresponding author.
Email: tmonroewhite@berry.edu

This PDF file includes:

Supplementary text
Figure S1 to S7
Tables S1 to S2
SI References
Legends for Datasets S1 to S11

Other supplementary materials for this manuscript include the following:

Datasets S1 to S11

Supplementary Information Text

Accompanying website with additional methodological details: https://sciencebias.uni.lu/app/.

Definitions

Joint probability is the proportion of articles for each racial group, gender, and topic, where

$$\boldsymbol{jp = P(r, g, t)},$$

with *r*: racial group, *g*: gender and *t*: topic. In this way, the sum of the joint probability of all articles, races, and genders equals 1.

Marginal probability by group is the proportion of each topic by racial group and gender, where

$$\boldsymbol{mpg = \frac{P(r, g, t)}{P(r, g)} = \frac{jp}{P(r, g)}},$$

Each racial group and gender sums to 1 in *mpg*.

Marginal probability by topic is the proportion of each racial group and gender by topic, where

$$\boldsymbol{mpt = \frac{P(r, g, t)}{P(t)} = \frac{jp}{P(t)}}$$

The marginal probability by topic sums to one for each topic.

The *over or underrepresentation* by racial group and gender is, proportionally, how much more or less present a group is with what is expected at random, given the overall share of that racial group and gender in the full dataset.

$$\boldsymbol{x = \frac{\frac{P(r, g, t)}{P(t)}}{P(r, g)} - 1 = \frac{mpt}{P(r, g)} - 1}$$

Data Sources

The bibliometric database used for our analysis is Clarivate Analytics' Web of Science (WOS). In addition to country information and citation indicators, we used given names of authors to infer their probably gender, and their family names to infer their probable race. Field and subfield classification is that of the National Science Foundation (*1*). To build topics, we use titles, keywords, and abstracts. Given that before 2008 first names are not provided by WOS, we restrict our analysis to the period 2008-2019 in order to infer author gender. Racial categories are a social construct that varies by country. Therefore, our analysis is limited to the United States. The information provided by the 2010 US Census on family names and their distribution by race and Latinx origin was used for racial inference (see below).

Race and Gender Inference

The method used for the racial inference based on names can be found in (*2*). We briefly highlight its main characteristics below. Our work is guided by the words of Tukufu Zuberi (*3*): "The racialization of data is an artifact of both the struggles to preserve and to destroy racial stratification." Similarly, Buolamwini and Gebru's work also showed the dangers of algorithmic bias (*4*). Therefore, we focused on different possible methodologies to infer race from names, and the

potential biases they carry. Family names from the 2010 US census (*5*) were used as a primary source of information on racial categorizations. We also attempted to use data on the distribution between given names and race from mortgage data (*6*). In all, in our attempt to minimize bias, we developed several strategies: 1) collapsing probability distribution into a single metric, 2) using thresholds, 3) using first names, and then given names, and 4) mixing given and family names' probabilities.

Ultimately, we found that the distribution by race when retaining names with >x% probability of belonging to a racial group—i.e., thresholding—is strongly affected by x. Therefore, the 'informativeness' of family names is heavily dependent on the racial categorization itself. This result is deeply related with the history of US racial classification and naming conventions. Descendants of enslaved Africans have historically shared surnames with their White enslavers. Disambiguating Black from White names therefore is more challenging in the US context, reinforcing the need to understand historical context when developing race-based algorithms like ours. Our main conclusion is that thresholds can heavily *underestimate* racialized groups, especially the Black population. One way to avoid this bias is to use the full distribution across racial groups, even if this complicates future analyses.

Topic Modeling

In order to compare the robustness of the LDA, we developed an experimental approach. The randomness of LDA can be controlled by a random seed. Each random seed provides different results for the same model and dataset. A non-robust model would provide very different results with each iteration. A robust model should only display minor changes. The latter guarantees that the results observed are not the product of chance. To test for this, we ran the LDA model for Social Sciences, Humanities, and Professional Fields ten times with different random seeds. By comparison, we used the pre-trained model on Health to predict the data from Social Sciences, and generated a completely random case using a Dirichlet distribution with the same dimensions. Importantly, the order of topics in the LDA is not fixed. What may be referred to as topic #2 in one model may be topic #17 in another. Therefore, we used a column-permutation invariant metric of distance to compare models.

The result of the LDA model was a matrix of dimensions NxT, with N articles and T topics. The proposed measure of distance first gets the L2 norm of each article. This assigns a single value for every article and, for each run of the model, a vector. We then use these vectors to compare the similarity between models using cosine similarity. The results (see figure S7) illustrate that all runs using random seeds are very similar between each other, while the results using a model trained on a different dataset and the random case are both very different to all other cases. This result validates our model and demonstrates that the results are not a simply a product of chance.

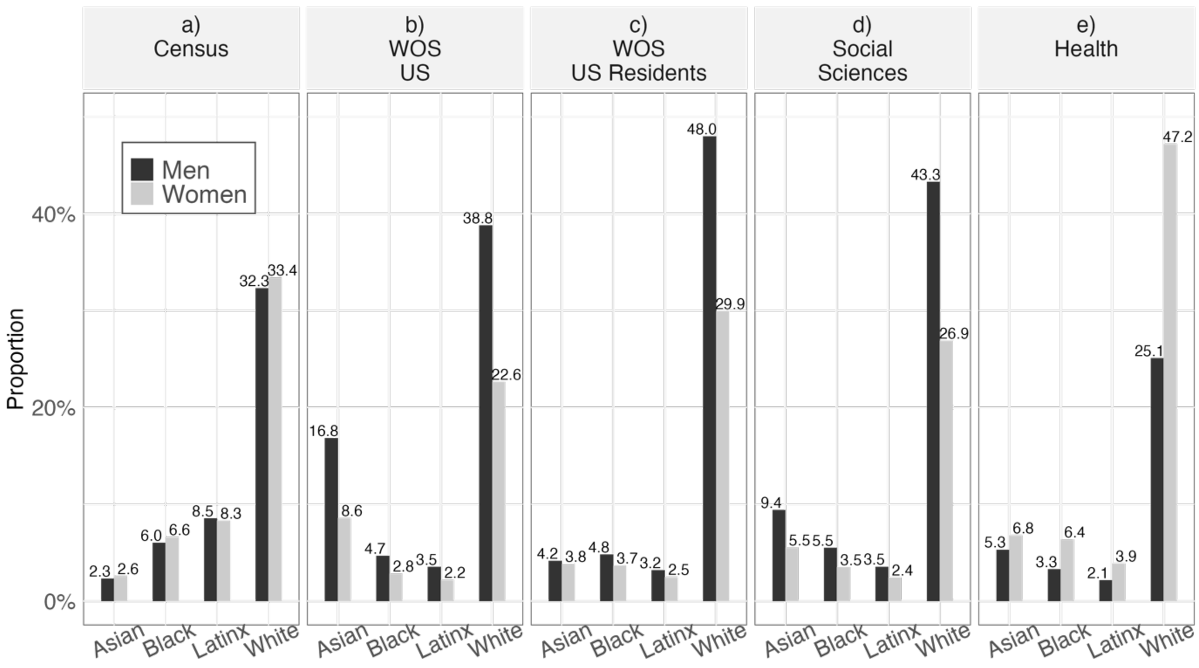

Fig. S1. Distribution of the population by race & gender a) in U.S. Census 2010, b) U.S. first authors of papers indexed in Web of Science 2008-2019, c) U.S. first authors normalized by the proportion of U.S. permanent residents who hold a doctorate, d) U.S. first authors of articles from Social Sciences, Humanities and Professional Fields and e) U.S. first authors of articles from the Health discipline. Racial categories from the census corresponding to 'AIAN' and 'Two or more' were excluded from the racial inference due to lack of data.

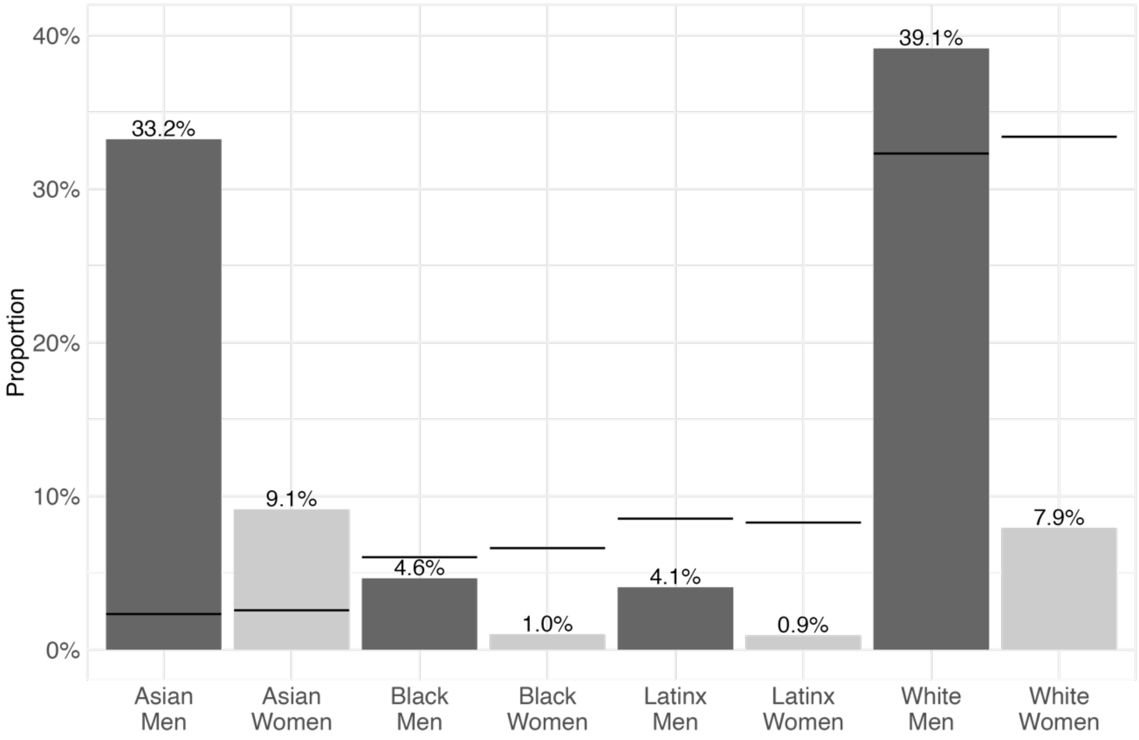

Fig. S2. Distribution by race and gender of US authors in Engineering and Technology, 2008-2019.

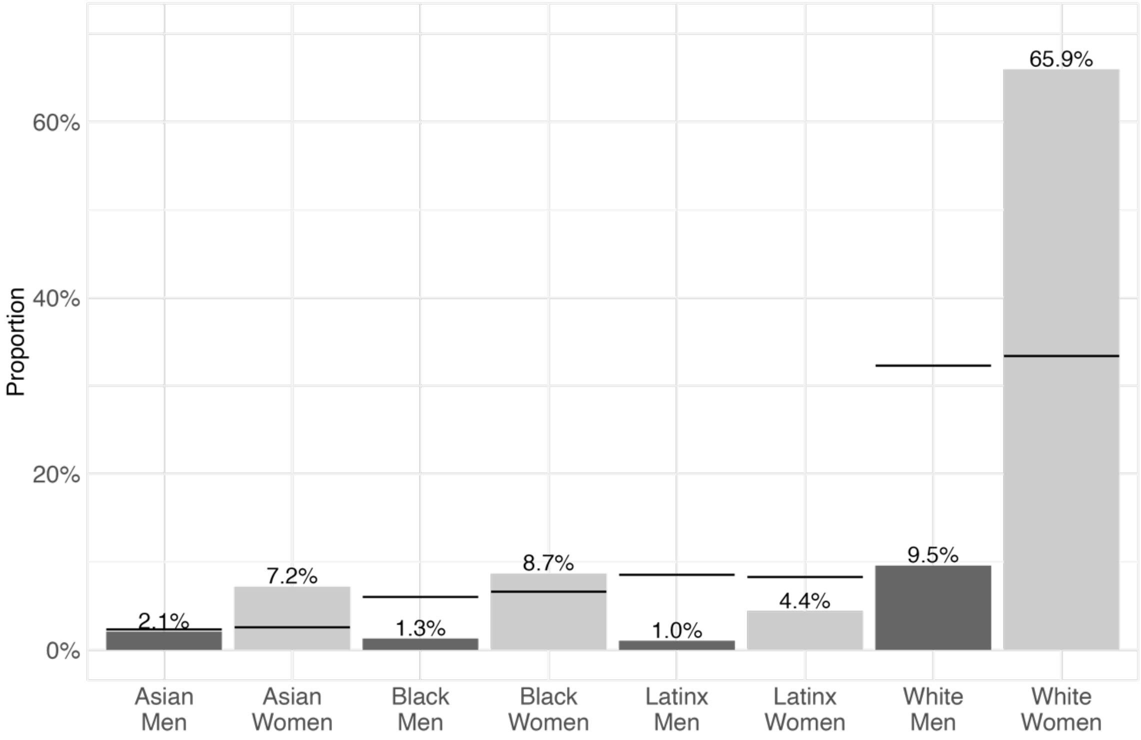

Fig. S3. Distribution by race and gender of US authors in Nursing, 2008-2019.

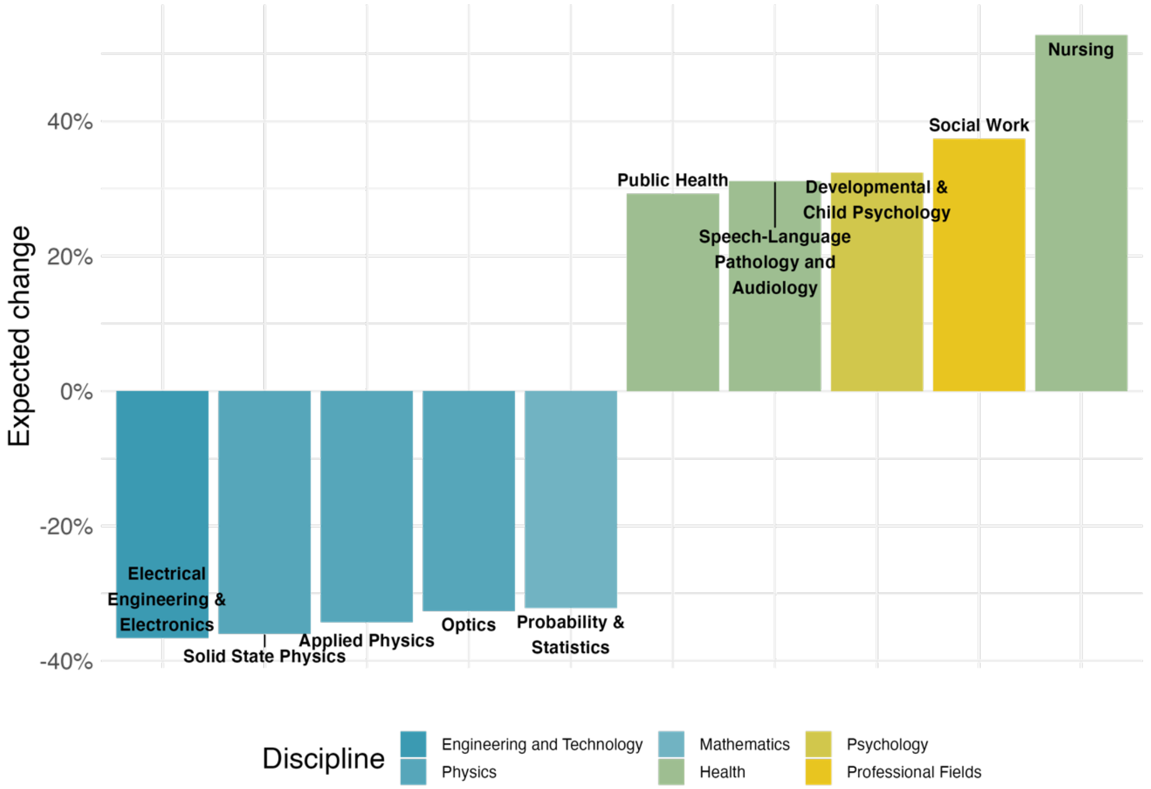

Fig. S4. Counterfactual analysis, US authors, 2008-2019. Assuming that authors are equally productive along their career, the y-axis represents the expected cumulative change in the number of papers per speciality, if the proportion of authors by race & gender would be that of the 2010 US Census. The x-axis shows the 5 specialities with highest decreases and increases.

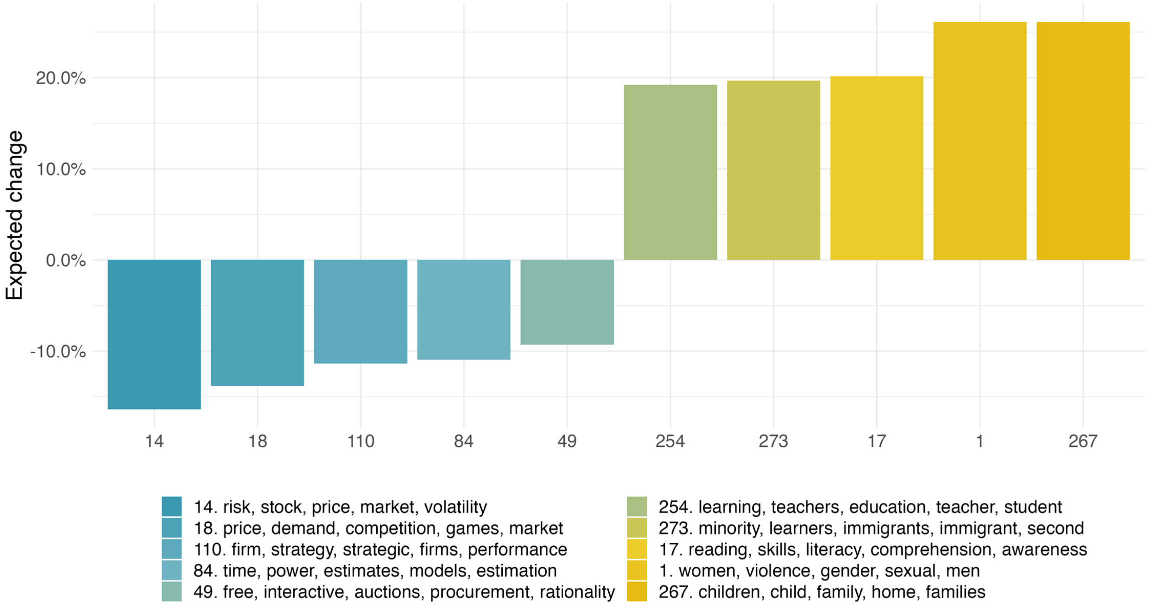

Fig. S5. Counterfactual analysis, Social Sciences, Humanities and Professional Fields, US 2008-2019. Assuming that authors are equally productive along their career, y-axis represents the expected cumulative change in the number of papers per topic if the proportion of authors by race & gender would be that of the 2010 US Census, for the 5 topics with the highest decrease and increase. This does not assume a change in the proportion of the Social Sciences, Humanities and Professional Fields disciplines in the overall distribution.

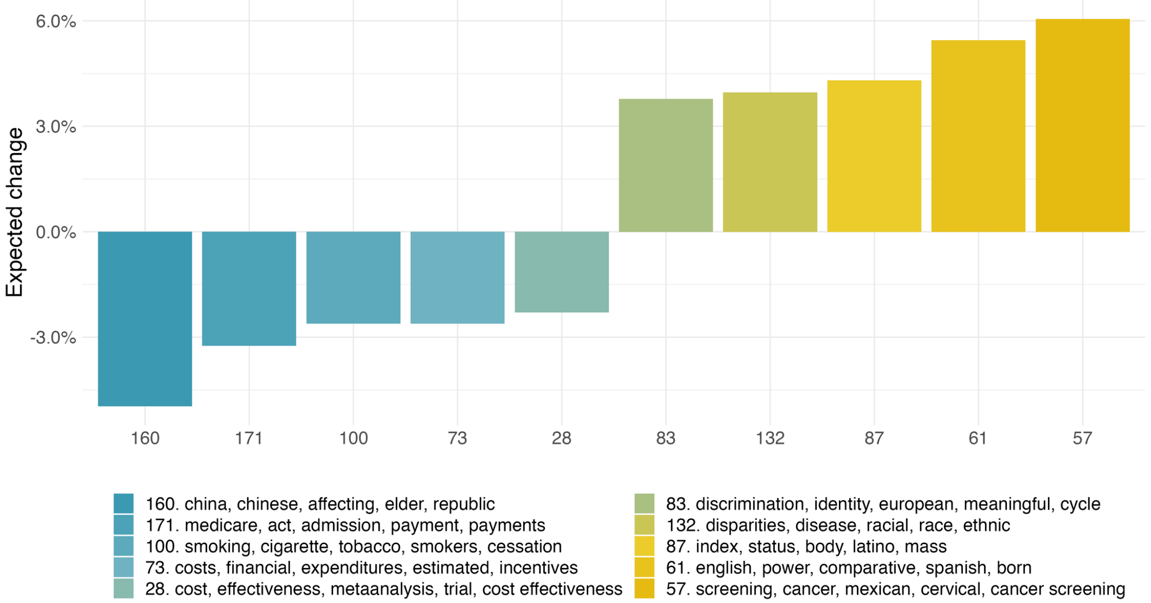

Fig. S6. Counterfactual analysis, Health discipline, US 2008-2019. Assuming that authors are equally productive along their career, the y-axis represents the expected cumulative change in the number of papers per topic if the proportion of authors by race & gender would be that of the 2010 US Census, for the 5 topics with the highest decrease and increase. This does not assume a change in the proportion of the Health discipline in the overall distribution.

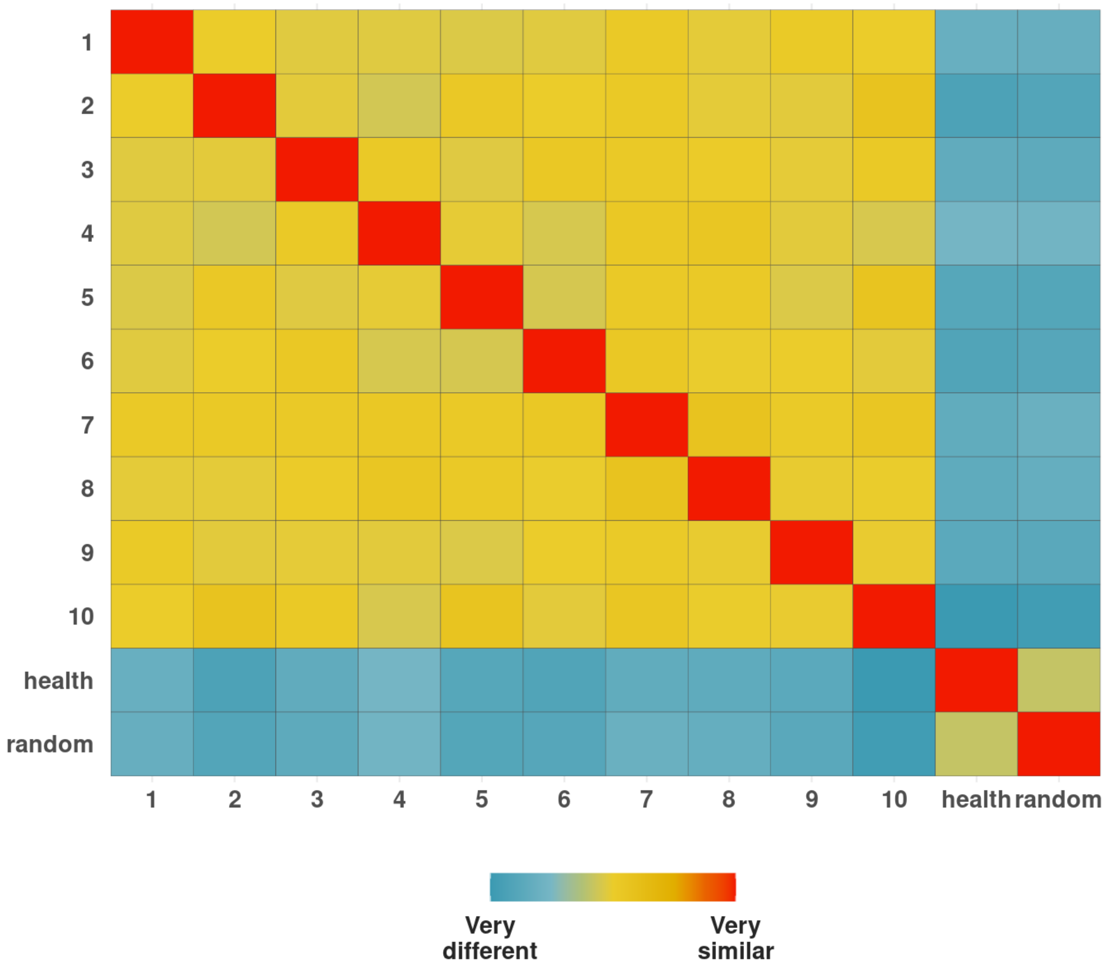

Fig. S7. Cosine similarity between multiple runs of the LDA model. Models 1 to 10 represent a model trained on the same social science dataset, with different random seeds. Model ‘health’ is a model with the same number of topics trained on the health dataset. All models were used to predict the same cases (social science data), resulting in a distribution of topics by document. The ‘random case’ uses a Dirichlet distribution that replicates the dimensions of the results. All results are compared using L2 norm and cosine similarity. All models trained on the same data showed a similar behavior, while the to control groups show a very different pattern. This confirms that the LDA results are not a product of chance, but reflect the properties of the database.

Table S1. Most frequent words and labels for a selection of topics in Social Sciences Humanities and Professional Fields.

Topic	Top Words	Label
1	women, violence, gender, sexual, men	gender-based violence
14	risk, stock, price, market, volatility	stocks
17	reading, skills, literacy, comprehension, awareness	literacy
18	price, demand, competition, games, market	market
28	states, united, united states, community, communities	US communities
36	african, democracy, sub, income inequality, saharan	Africa
51	political, politics, identity, participation, latin	political-identity
77	gay, transgender, lesbian, bisexual, lgbt	LGBT
83	religious, religion, human rights, socialization, church	religion
110	firm, strategy, strategic, firms, performance	firms
160	race, racial, black, white, discrimination	racial-discrimination
201	argentina, ghana, pension, nonresponse, oaxaca	Argentina Ghana
204	model, models, selection, search, modeling	modelling
218	american, cultural, culture, seeking, african american	African-American culture
251	language, life, english, chinese, reliability	language
254	learning, teachers, education, teacher, student	learning
267	children, child, family, home, families	families
273	minority, learners, immigrants, immigrant, second	immigrant
297	product, consumer, consumers, brand, products	consumer

Table S2 Most frequent words and labels for a selection of topics in Health.

Topic	Top Words	Label
11	women, health, pregnancy, pregnant, reproductive	pregnancy
14	human, cell, cells, expression, stem	cells
16	african, american, african american, south, americans	African American
57	screening, cancer, mexican, cervical, cancer screening	Mexican
61	english, power, comparative, spanish, born	English-Spanish
66	men, hiv, gay, msm, sex	gay men
72	response, muscle, activation, resistance, dynamic	muscle
73	costs, financial, expenditures, estimated, incentives	costs
83	discrimination, identity, european, meaningful, cycle	discrimination
87	index, status, body, latino, mass	Latinx body
92	education, students, learning, educational, suicide	education
104	sexual, risk, sex, prevention, hiv	STD
116	men, lung, cancer, lung cancer, prostate	men cancer
119	beta, aging, protein, regulation, mice	protein
132	disparities, disease, racial, race, ethnic	racial disparities
160	china, chinese, affecting, elder, republic	China
187	community, engagement, prevention, communities, focus	community
196	nurses, nursing, work, practice, care	nursing

Dataset S1 (separate file). Distribution by field. Probability of specialty, joint probability, marginal by specialty, marginal by group, over/underrepresentation, median and mean citations.
Dataset S2 (separate file). Joint probability of race, gender and topic, Social Sciences Humanities and Professional Fields.
Dataset S3 (separate file). Marginal probability of race and gender by topic, Social Sciences Humanities and Professional Fields.
Dataset S4 (separate file). Marginal probability of topics by race and gender, Social Sciences Humanities and Professional Fields.
Dataset S5 (separate file). Over/under representation of race and gender groups by topics, Social Sciences Humanities and Professional Fields.
Dataset S6 (separate file). Mean, median and standard deviation of citations by topic, race and gender, Social Sciences Humanities and Professional Fields.
Dataset S7 (separate file). Joint probability of race, gender and topic, Health.
Dataset S8 (separate file). Marginal probability of race and gender by topic, Health.
Dataset S9 (separate file). Marginal probability of topics by race and gender, Health.
Dataset S10 (separate file). Over/under representation of race and gender groups by topics, Health.
Dataset S11 (separate file). Mean, median and standard deviation of citations by topic, race and gender, Health.

SI References

1. K. Hamilton. Subfield and Level Classification of Journals (CHI Report No. 2012-R). Cherry Hill, NJ: CHI Research (2003).

2. D. Kozlowski, D.S Murray, A. Bell, W. Hulsey, V. Larivière, T. Monroe-White, C.R. Sugimoto. Avoiding bias when inferring race using name-based approaches. Proceedings of the 18th International Conference on Scientometrics & Informetrics, pp. 597-608. arXiv preprint arXiv:2104.12553 (2021).

3. T. Zuberi. Thicker than blood: How racial statistics lie. U of Minnesota Press (2001).

4. J. Buolamwini, & T. Gebru. Gender shades: Intersectional accuracy disparities in commercial gender classification. In Conference on fairness, accountability and transparency (pp. 77-91). PMLR (2018, January).

5. U.S. Census Bureau. Frequently Occurring Surnames from the 2010 Census. The United States Census Bureau (2016). https://www.census.gov/topics/population/genealogy/data/2010_surnames.html

6. K. Tzioumis. Demographic aspects of first names. Scientific data, 5, 180025 (2018).
